# A Theoretical Study on Friction of Macroscale Patterned Surfaces: Implications for Scaling Up Superlubricity

**DOI:** 10.1021/acsami.5c16288

**Published:** 2025-09-25

**Authors:** Viet Hung Ho, Melisa M. Gianetti, Ahmed Uluca, Aaron D. Sinnott, Bjørn Haugen, Graham L. W. Cross, Astrid S. de Wijn

**Affiliations:** † Department of Mechanical and Industrial Engineering, 8018 Norwegian University of Science and Technology (NTNU), 7491, Trondheim, Norway; ‡ School of Physics and CRANN, 8809 Trinity College Dublin, Dublin 2, D02 W085, Ireland

**Keywords:** structural superlubricity, discrete element method, contact mechanics, two-dimensional coating

## Abstract

“Structural superlubricity”, a state of frictionless sliding between crystalline surfaces, has been observed at the nanoscale and microscale. However, achieving it at the macroscale requires further investigation. Inspired by recent experimental studies, we theoretically examine the friction behavior of macroscale patterned surfaces composed of microscale bumps coated with superlubricious two-dimensional materials. We performed numerical simulations with the discrete element method. The Hertz contact model, along with a modified tangential Mindlin contact model, is employed to capture the nonlinear relationship between the coefficient of friction and normal load. Our results reveal that the friction behavior is significantly influenced by the radius of the microscale bumps, the durability of the coating, and the elasticity of the surface, and we show how those can be tuned to improve friction properties. Additionally, we analytically investigate the deformation mechanisms of the surface structure and derive scaling laws for parameters and the breakdown of superlubricity. The simulation results show strong agreement with the analytical derivations of power laws for scaling of various quantities with the total macroscopic load. Finally, we examine imperfect conditions by investigating how height variations impact frictional performance.

## Graphical abstract

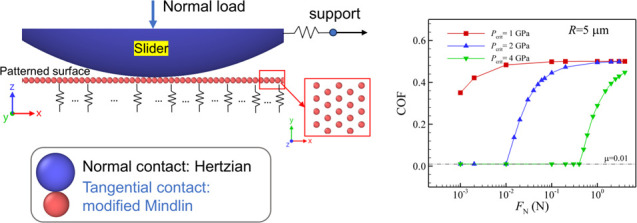

## 1 Introduction

It is estimated that over 20% of the world’s total energy consumption is lost to friction,^1^ and another 3% due to wear. Therefore, reducing friction and wear is important for both economic and environmental sustainability. The majority of wear, and a significant portion of the friction, is due to solid-on-solid contacts. Over the past decades, numerous studies have been performed to understand the nature of solid friction at the nanoscale with the objective of achieving ultralow friction.

An important phenomenon in the study of friction is superlubricity. In the engineering context, this usually refers to cases where the friction coefficient is 0.01 or lower. Since macroscale surfaces in the real world are typically rough and only come into contact at nano- or microscale contact asperities, any macroscopic superlubricity must originate from low friction at small scales. With the advent of AFM, there has been some considerable effort expended to finding possible mechanisms of low friction in nanoscale solid-on-solid contacts. One such mechanism that has attracted a lot of attention is structural superlubricity,^2–11^ which is (nearly) vanishing friction between two atomically flat incommensurate crystalline contacts. It was first predicted in a theoretical study by Hirano et al.^2^ Low friction due to structural incompatibility has been observed across a wide range of interfaces, first reported by Hirano et al.^12^ Later experiments by Dienwiebel et al.^3^ using flat, rotated graphite flakes sliding on flat, graphitic substrates did reach engineering superlubricity. Subsequently, structural superlubricity has been found in various two-dimensional (2D) materials, including graphene,^13–16^ molybdenum disulfide (MoS_2_),^17,18^ and transition-metal carbides and nitrides (MXenes),^19^ graphite,^3,20,21^ diamond-like carbon (DLC),^22^ and heterostructures.^23–28^ It has been achieved at the nanoscale^3,4,29,30^ and microscale.^14,24,31–33^

In contrast, experiments on the macroscale using these materials usually show high coefficients of friction (COF). The breakdown of structural superlubricity results from different mechanisms related to size, such as elastic relaxation,^34^ and the nonideal nature of the macroscopic interfaces, including edge effects,^23,35,36^ surface roughness,^37–39^ puckering effects,^40,41^ surface defects,^42–44^ and adsorbed layers or contaminants.^45–50^ All of these break the special conditions required for structural superlubricity. Consequently, achieving superlubricity in the engineering sense at the macroscale using structural superlubricity remains a challenge. Nevertheless, there are a few experimental studies that provide hope and have demonstrated macroscale superlubricity based on structural superlubricity,^51–55^ at least for some time. For instance, Androulidakis et al.^54^ experimentally showed that the coefficient of friction of a macroscale size graphitic sample could be tuned and reach a superlubricity regime through strain engineering. This finding is in good agreement with the theoretical study,^56^ but the approach still depends on difficult to achieve ideal conditions.

Using a different approach with patterned surfaces, Li et al.^52^ reported an ultralow coefficient of friction (COF) in magnitude of 10^–3^ between a steel ball and graphene/MoS_2_ coated, laser textured steel substrates, containing microasperities. Recently, Aymard et al.^57^ introduced a novel experimental strategy for designing metainterfaces that can control macroscopic friction. These meta-interfaces contain multiple microscale spherical bumps. By optimizing the height, radius, and distribution of these bumps, the authors were able to engineer the friction behavior of the interfaces. However, without theoretical understanding of the role of patterning, engineering such superlubric interfaces is a slow trial-and-error process.

Inspired by the experimental studies conducted by Li et al.^52^ and Aymard et al.,^57^ we numerically and analytically investigate the friction behavior of a macroscale patterned surface structure composed of multiple microscale bumps with engineering superlubricity, which could, for example, be achieved by coating them with structurally superlubricious 2D materials. Our study employs the Hertz contact model along with our modified tangential Mindlin contact model, which can capture the failure of the coating under high contact pressure. The effects of the radius of the microscale bumps, the durability of 2D coating layer, the elasticity of the surface, and fabrication defects on the macroscale friction behavior of the patterned surface are investigated. In addition, we also discuss the deformation mechanisms of the surface structure and the combination of them. The simulation results strongly align with the analytical derivations of the scaling laws of various quantities with respect to the macroscopic load.

## 2 Simulation Models and Methods

Figure 1 illustrates our model of a macroscale patterned contact structure with the surfaces coated with 2D materials while subjected to a sliding test. The pattern consists of microscale granular particles (red color), which represent microscale elastic bumps on a surface, arranged as a hexagonal lattice with an interparticle distance (*p*) of 3 μm. The radius (*R*) of the particles can be varied, and we will discuss this effect on the friction behavior in the following section. The orientation of the particle layout is fixed. To represent the elasticity of the patterned surface, each particle is tethered to its initial position via linear springs in the *x*-, *y*-, and *z*-directions (*K*_*x*_, *K*_*y*_, and *K*_*z*_), but they remained unlinked and independent of each other. The smooth sphere slider (blue in Figure 1), which has a diameter of 10 mm, is subjected to a constant normal load in the vertical (*z*-) direction. It is connected to a supporting particle by a spring along the *x*-direction. The slider interacts elastically with the particles, but the elastic interactions between the particles were not considered. We also examined the effect of the elastic interaction between particles on COF and found this effect is negligible. Further details are provided in the Supporting Information. The simulations were implemented in LAMMPS^58,59^ using the Granular package with our own modifications. Given the type of interaction and length scales that we describe, our simulations could be considered either extremely coarse-grained molecular dynamics (MD) or a fairly simple discrete element method (DEM). To model the loss of kinetic energy to the environment, the system has a viscous damping with a damping time of 1 μs. A time step of 10^–5^ μs is used. The system was first equilibrated for 20 μs, with the normal load applied to the slider. Next, the supporting particle was pulled at a constant velocity of 1 m/s for 100 μs. The friction force was calculated as the average force in the spring connecting the slider to the support particle. In our simulations, Young’s modulus was set to 100 GPa and Poisson’s ratio to 0.3. We selected these values because they are representative in order of magnitude for many engineering materials, including steel, which was used in the previous experimental study.^52^ It is worth noting that the relation between the elastic behavior of the asperities and surface is nontrivial. Bulk properties and surface properties on the microscale are not necessarily the same; the geometry of the bumps and how they are attached to the surface can be varied, and any surface treatment could affect the material. Consequently, there is no direct obvious link between the type of elasticity and the parameters, i.e., the Young’s modulus and the spring constants. Therefore, these quantities are treated as independent of our analysis. The density of granular particles in the patterned surface was set to 2.2 g/cm^3^. The mass of the slider was set as 9.2 × 10^–11^ g. For the elastic Hertz and Mindlin contact models (described below), the damping prefactor coefficient was 10^–3^ (μs × μm)^−1^ and 10^–4^ (μs × μm)^−1^, respectively. The spring stiffness was set to 1.27 × 10^4^ N/m for the in-plane *K*_*x*_ and *K*_*y*_ and 1.1 × 10^5^ N/m for *K*_*z*_. The estimation of spring stiffness is provided in the Supporting Information.

**Figure 1:**
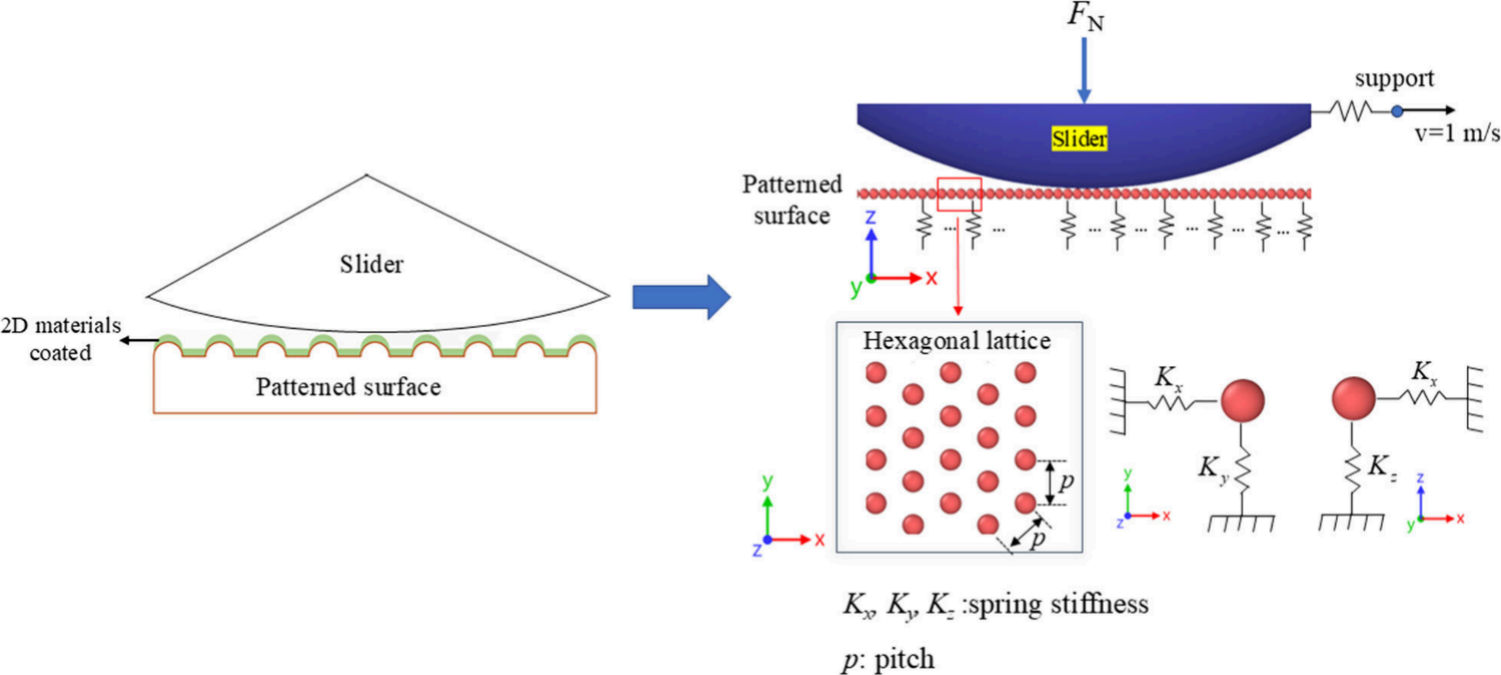
A macroscale patterned surface structure coated with 2D materials subjected to a sliding test. The surface consists of granular particles (red) presenting microscale bumps arranged in a hexagonal lattice. The large slider (blue) has a gently curved surface and is subjected to a constant normal load. The superlubric coating is modeled through a very low friction coefficient, described by our modified Mindlin model (see eq 1).

The contact between surface particles and the slider was modeled using the Hertz model for the mechanics in the normal direction^60^ and our modified Mindlin model for tangential direction. The superlubric coating likely has different elastic properties from the bulk material underneath, but Hertz’s theory can still be applied, as the coating thickness is of the order of a nanometer, while the contact size in our system is typically of the order of microscale. The Mindlin model is suitable for micrometer-scale contacts between curved asperities. At smaller scale, more complex friction may appear.^61^ It is noted that there is a distinction between engineering superlubricity and structural superlubricity. In structural superlubricity, scaling laws are very different than they are in regular contacts, but when superlubricity breaks down due to various reasons such as contamination or elasticity, typically the regular linear contact area scaling is recovered but potentially with a small friction coefficient. Therefore, in our model, we consider engineering superlubricity to be a reasonable approximation for nonideal contacts with structurally superlubricious coatings. For the tangential direction, we also considered the failure of the superlubricious coating. Previous experimental studies have demonstrated that the 2D material coating layer can exhibit structural superlubricity under high contact pressures,^14,53,62^ reaching up to several gigapascals.^62^ However, as the contact pressure exceeds a critical threshold, the failure of the 2D material coating leads to a breakdown of superlubricity^62–64^ and a drastic increase in friction by orders of magnitude, which can be attributed to the rupture of the graphene layer.^63^ Therefore, the durability of the coating plays a crucial role in the superlubricity of the system. In the Mindlin no-slip tangential contact model,^65^ when two particles start sliding relative to each other, the friction force is linearly related to normal load, as the COF is independent of the contact pressure. In order to capture the pressure dependence of friction coefficient and especially the breakdown of superlubricity, we propose a modified Mindlin contact model in which the tangential force is calculated as

1 $$\{\begin{array}{cc} F_{t}=-\min\left(\mu_{1}f_{N},||-k_{t}a\xi +F_{\mathrm{damp}}||\right)t & \mathrm{if},P_{\max}<P_{\mathrm{crit}}\\ F_{t}=-\min\left(\mu_{2}f_{N},||-k_{t}a\xi +F_{\mathrm{damp}}||\right)t & \mathrm{if},P_{\max}\geq P_{\mathrm{crit}} \end{array}$$

Here, *a* is the contact radius, ξ is the tangential displacement accumulated during the duration of contact, **t** is the unit vector in the tangential direction, and **F**_damp_ is the tangential damping force which is given by **F**_damp_ = −η_*t*_ **v**_*t*,*rel*_ where η_*t*_ is tangential damping prefactor and **v**_*t*,*rel*_ is relative tangential velocity at the contact point. The coefficient *k*_*t*_ is defined as *k*_*t*_ = 8*G*_*eff*_. Here, *G*_*eff*_ is effective shear modulus calculated as $G_{eff}={\left(\frac{2-\nu_{i}}{G_{i}}-\frac{2-\nu_{\mathrm{slider}}}{G_{\mathrm{slider}}}\right)}^{-1}$ , with *G*_*i*_, *G*_slider_ being the shear moduli of particles *i* in the surface and slider, respectively. The shear modulus is related to the Young’s modulus and the Poison’s ratio as *G* = *E*/2­(1 + *v*). *f*_N_ is the normal force and *P*_max_ is the maximum Hertzian contact pressure on each microscale contact, which are calculated as

2 $$f_{N}=\frac{4}{3}E_{\mathrm{eff}}R_{\mathrm{eff}}^{1/2}\delta^{3/2},\mathrm{and},P_{\max}={\left(\frac{6f_{N}E_{\mathrm{eff}}^{2}}{\pi^{3}R_{\mathrm{eff}}^{2}}\right)}^{1/3}$$

where *E*_eff_ and *R*_eff_ are effective Young’s modulus and effective radius, respectively; and δ is the normal elastic deformation, which in the DEM is often referred to as the overlap distance between two particles. The effective Young’s modulus is given by $E_{\mathrm{eff}}={\left(\frac{1-\nu_{i}^{2}}{E_{i}}+\frac{1-\nu_{\mathrm{slider}}^{2}}{E_{\mathrm{slider}}}\right)}^{-1}$ where *E*_*i*_, *E*_slider_ are Young’s moduli and *v*_*i*_, *v*_slider_ are Poisson’s ratios; and the effective radius is $R_{\mathrm{eff}}={\left(\frac{1}{R_{i}}+\frac{1}{R_{\mathrm{slider}}}\right)}^{-1}$ with *R*_*i*_ and *R*_slider_ being radius of the surface particle and slider, respectively.

In eq 1, *P*_crit_ is defined as a critical pressure in which failure of the 2D coating layer occurs, and μ_1_ and μ_2_ are COF at two different friction regimes. The basic idea of the modified Mindlin model is as follows:

- First, the maximum Hertzian contact pressure (*P*_max_) is calculated at each microscale contact.
- If the calculated *P*_max_ is smaller than the *P*_crit_, then coating layer is retained, and the microscale contact remains in the superlubric regime.
- Otherwise, if *P*_max_ exceeds *P*_crit_, superlubricity breaks down, resulting in a high COF. This process can be reversible. While damage to the coating is, in principle, not reversible, in experiments it has been observed that superlubricity can be restored when the load is reduced.^52^

We have implemented this modified Mindlin model in the Granular package for LAMMPS. To verify our code, we have checked the sliding between two individual particles. More details of this can be found in the Supporting Information. In the rest of our study, we use 0.01 and 0.5 for μ_1_ and μ_2_, respectively.

## 3 Deformation Mechanisms and Analytical Derivation for Scaling Laws

Before presenting the simulation results, we first elucidate the deformation mechanisms of the patterned surfaces and analytically derive power laws for scaling of various quantities with a total macroscopic load. As mentioned in the previous section, the surface structure is modeled with elastic granular particles tethered by springs to their initial positions. As shown in Figure 2, we find that our model has two mechanisms which affect the vertical displacement and force at the contact: (i) the deformation of the embedding surface material (spring mechanism, denoted by S) and (ii) the direct deformation of the contacts between the particles and slider (overlap mechanism, indicated by O). The deformation of the surface along the *z-*direction depends on the spring stiffness *K*_*z*_, while the deformation of the particles at the microscale contact is accounted for using Hertzian contact mechanics. In the limit of high Young’s modulus or soft springs, the spring compression mechanism dominates, while in the opposite limit, the material deformation (overlap mechanism) prevails. Our simulations reveal a combination of both mechanisms. Since the slider is significantly larger than the particles, particle motion along the *z-*direction is more dominant compared to the *x-* and *y-*directions. Therefore, we may assume that the contact normal is always pointing in the *z-*direction and all deflection occurs in the *z-*direction. In this case, we can use the geometry of the slider with radius *R*_slider_ to approximate the deflection in each microscopic contact as a function of the distance *r* ≪ *R*_slider_ to the center of the macroscopic contact as

3 $$\delta \left(r\right)\approx \frac{a^{2}-r^{2}}{2R_{\mathrm{slider}}}$$

where *a* ≪ *R*_slider_ is the radius of the macroscopic contact. This deflection can originate from deformation of the particles or from the compression of the tethering springs. We now calculate the forces needed to achieve this deflection in both mechanisms, while keeping track of power law scaling with the macroscopic contact radius *a*. We also track scaling with the effective radius parameter *R*_eff_, which is dominated by the small particle radius; i.e., *R*_eff_ ≈ *R*.

**Figure 2:**
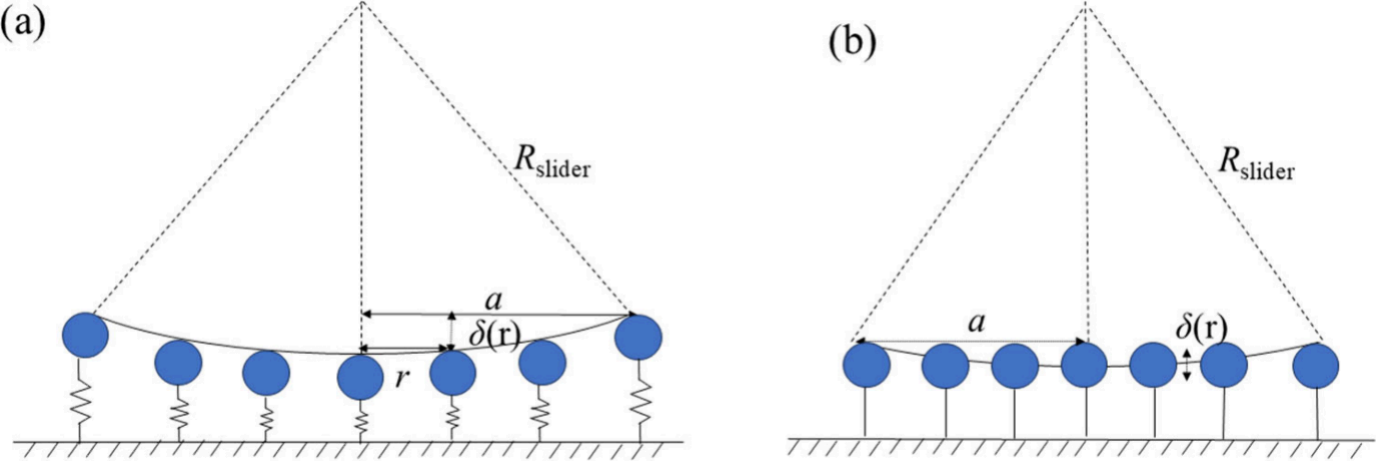
Deformation of the contact surface under normal load. (a) Spring compression mechanism and (b) overlap mechanism.

In the limit of stiff springs and soft materials, the surface pattern can be treated as rigid, while the local contact compliance given by Young’s modulus of the particles is finite. In this case, all deformation is due to local Hertzian contacts and the deflection (or overlap as it is referred to in the DEM and eq 2) and normal force *f*_N_^(O)^(*r*) in a particle contact at position *r* are related through

4 $$\left|\delta \left(r\right)\right|\propto {\left(\frac{f_{N}^{\left(O\right)}{\left(r\right)}^{2}}{E_{\mathrm{eff}}^{2}R_{\mathrm{eff}}}\right)}^{1/3}$$

where *E*_eff_ and *R*_eff_ are the effective elastic modulus and effective radius, respectively. From eq 4, this yields an expression for the force in each contact

5 $$f_{N}^{\left(O\right)}\left(r\right)\propto {\left|\delta \left(r\right)\right|}^{3/2}E_{\mathrm{eff}}R_{\mathrm{eff}}^{1/2}$$

In the limit of soft springs and stiff materials, it is only the springs that deform, and the force in each contact *f*_N_^(S)^(*r*) is linearly dependent on the deflection; i.e.,

6 $$f_{N}^{\left(S\right)}\left(r\right)=-K\delta \left(r\right)$$

To obtain the total load, we sum up over the *N* microscopic contacts that make up the macroscopic contact and approximate the sum by a normalized integral over the area of the contact

7 $$F_{N}^{\left(O,S\right)}\left(r\right)=\sum f_{N}^{\left(O,S\right)}\left(r\right)\approx \frac{N}{\pi a^{2}}\int_{0}^{a}2\pi rf_{N}^{\left(O,S\right)}\left(r\right)dr$$

Substituting eqs 5 and 6, as well as eq 3, and performing the integrals, we find

8 $$F_{N}^{\left(O\right)}\propto a^{5}R_{\mathrm{eff}}^{1/2}$$

9 $$F_{N}^{\left(S\right)}\propto a^{4}$$

The maximum load on a microscopic contact is the load on a contact in the center, i.e., *f*_N_^(O,S)^(0). The scaling of this can also be obtained by combining eqs 5 and 6, with eq 3, which yields

10 $$f_{N}^{\left(O\right)}\left(0\right)\propto a^{3}R_{\mathrm{eff}}^{1/2}$$

11 $$f_{N}^{\left(S\right)}\left(0\right)\propto a^{2}$$

Combining these last two sets of eqs (eqs 8, 9, 10, and 11) and matching the scaling with *a* yields a scaling relationship between the macroscopic load and maximum microscopic load,

12 $$f_{N}^{\left(O\right)}\left(0\right)\propto {\left(F_{N}^{\left(O\right)}\right)}^{3/5}R_{\mathrm{eff}}^{1/5}$$

13 $$f_{N}^{\left(S\right)}\left(0\right)\propto {\left(F_{N}^{\left(S\right)}\right)}^{1/2}$$

The maximum pressure *P*_max_ in a microscopic Hertz contact is related to the microscopic load that it carries and the radius curvature *R*_eff_ through *P*_max_ ∝ *f*_N_^1/3^ *R*_eff_^–2/3^. Using this scaling and eqs 12 and 13, we find that the final scaling of the maximum pressure with load in the two limiting cases

14 $$P_{\max}^{\left(O\right)}\propto {\left(F_{N}^{\left(O\right)}\right)}^{1/5}R_{\mathrm{eff}}^{-3/5}$$

15 $$P_{\max}^{\left(S\right)}\propto {\left(F_{N}^{\left(S\right)}\right)}^{1/6}R_{\mathrm{eff}}^{-2/3}$$

We finally find the scaling of the transition load at which the system goes from superlubric (below the critical pressure) to nonsuperlubric sliding (above the critical pressure),

16 $$F_{\mathrm{trans}}^{\left(O\right)}\propto {\left(P_{\mathrm{crit}}^{\left(O\right)}\right)}^{5}R^{3}$$

17 $$F_{\mathrm{trans}}^{\left(S\right)}\propto {\left(P_{\mathrm{crit}}^{\left(S\right)}\right)}^{6}R^{4}$$

where we have also used that *R*_eff_ ∝ *R*.

We can also derive a scaling for the number of microcontacts, *N*, as this is proportional to the macroscopic apparent contact area. Using eqs 10 and 11, we find

18 $$N^{\left(O\right)}\propto a^{2}\propto {\left(F_{N}^{\left(O\right)}\right)}^{2/5}R^{-1/5}$$

19 $$N^{\left(S\right)}\propto a^{2}\propto {\left(F_{N}^{\left(S\right)}\right)}^{1/2}$$

## 4 Results and Discussion

In this section, we present the results of our numerical simulations and investigate the impact of material parameters, such as the critical pressure at which the coating fails, the elastic parameters, and the geometry and placement of the granular particles. We compare the results to analytical results derived in the previous section and link them to the experiments of Li et al.^52^

### 4.1 Effect of Critical Pressure

In this section, we focus on the friction behavior of the patterned surfaces under various loading conditions and critical pressures. Several values of critical pressures were considered, specifically 1, 2, and 4 GPa. These values are of the same order of magnitude as the durability of van der Waals layer materials coating layer reported in previous experimental studies.^53,62^ First, we demonstrate the local contact pressure at individual microscale contacts. Figure 3a shows snapshots of the maximum Hertzian contact pressure distribution at the local microscale contacts for different normal loads. Since the load is predominantly carried by the particles at the center, a high local contact pressure is obtained in this region. The values of *P*_max_ can be orders of magnitude higher than the apparent macroscopic pressure. As the normal load increases, there is an increase in the deformation of the surface along the *z*-direction, resulting in a larger macroscopic contact area and number of contacts. The estimated maximum macroscopic pressures are approximately 7.5, 26.2, 108.3, and 368.4 MPa under normal load of 0.001, 0.01, 0.1, and 1 N, respectively. Figure 3b,c presents the number of contacts and average maximum local contact pressure (average *P*_max_) as a function of the normal load.

**Figure 3:**
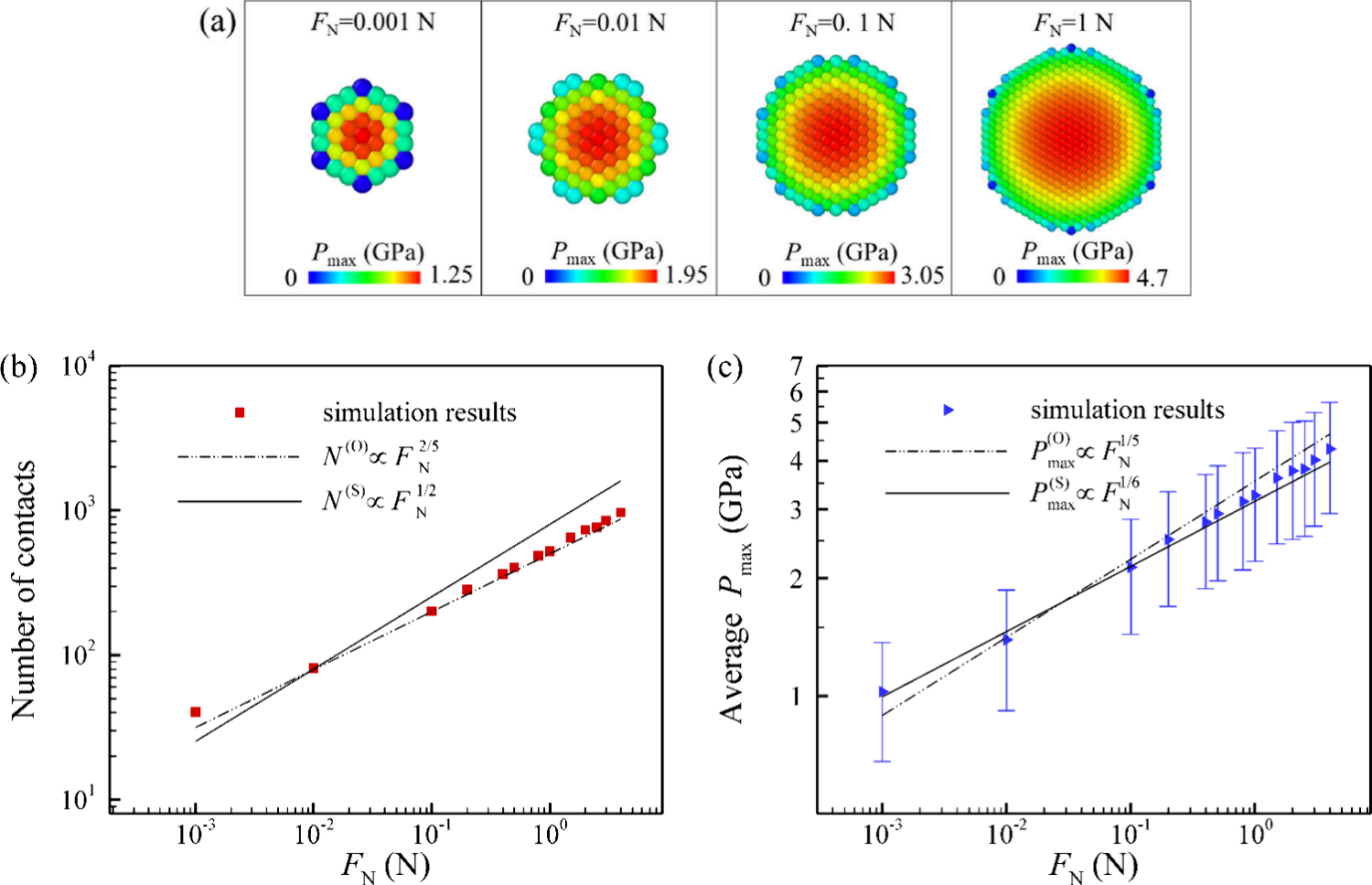
(a) Maximum Hertzian contact pressure (*P*_max_) at each individual microscale contact under different normal load (*F*_N_) ranging from 0.001 to 1 N. It is noted that the contact area and the corresponding contact pressure are localized at the apex of each particle, rather than across the entire particle. (b) Number of contacts and (c) average Hertzian contact pressure (average *P*_max_) as functions of normal load. The error bars in (c) represent the standard deviation. The radius of the particles is 5 μm. Higher pressures are observed at the microscale contacts. The derived theoretical power laws governing the scaling of the number of contacts (*N*) and *P*_max_ with normal load are bounded by two limiting cases: local Hertzian deformation due to overlap of contacts (denoted by O) when the springs are very stiff and spring compression (denoted by S) when they are very compliant. As can be seen, the simulation data lie between these two limiting power laws, most closely to the spring limit (S).

Figure 4 shows the nonlinear dependence of the friction force and the COF with normal load. We obtain a robust transitional point (*F*_trans_) where the friction behavior of macroscale patterned surface changes from superlubric (μ = 0.01) to a nonsuperlubric regime (μ > 0.01). If all local microscale contacts experience pressures which are smaller than the critical pressure, macroscale superlubricity is obtained. As the applied normal load increases, high contact pressures (*P*_max_ ≥ *P*_crit_) are observed at the contacts in the central region (Figure 3a), resulting in a high-friction zone. Meanwhile, the contacts in the outer region exhibit low pressure (*P*_max_ < *P*_crit_); thus, the outer areas exhibit superlubricity. The macroscopic frictional response is a combination of superlubricious microscale contacts at the outer region and high-friction contacts at the central region. We also find that for systems with higher critical pressures the transition point is obtained at a higher value of the normal load. As can be seen in Figure 3c, the average *P*_max_ of local microscale contacts exceeds 1 GPa already with normal load of 10^–3^ N. Therefore, with *P*_crit_ equal to 1 GPa, the surface structure has a high COF even at such low normal load. Under 10^–3^ N of normal load, the estimated macroscopic contact radius is approximately 8 μm, leading to a maximum macroscopic pressure of 7.5 MPa, which is significantly smaller than the pressure of the microscale contacts. As shown in Figure 4b, with the critical pressure equal to 2 and 4 GPa, the transitional point occurs at much higher normal loads, of approximately 0.015 and 0.5 N, respectively. Increasing the critical pressure from 2 to 4 GPa leads to an increase in *F*_trans_ by a factor of approximately 33. This dramatic change can be understood from the analytical expressions for the scaling that we derive in Section 3, where we demonstrate that the transition load force scales with the critical pressure with a power law with an exponent of between 5 and 6 (eqs 16 and 17). It is noteworthy that the pressure of individual local contact is calculated and compared with the critical pressure to determine the corresponding friction regime. Therefore, the transitional point depends on the *P*_crit_ but not on the friction coefficient parameters. Consequently, to maintain the superlubricity of the surface over a wider range of loading conditions, the durability of the coating layer is essential.

**Figure 4:**
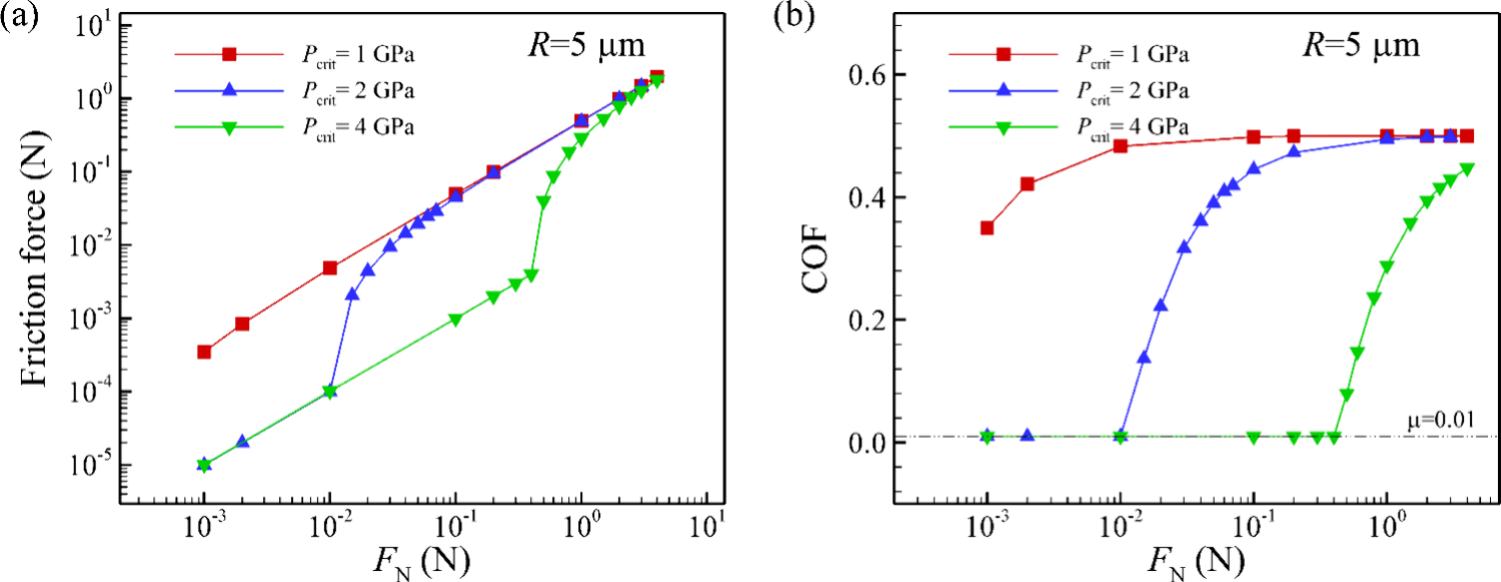
(a) Friction force and (b) COF of contact surface vs normal load using the Hertz and modified Mindlin contact model. The radius of the surface particles was set to 5 μm, and various values of critical pressure were examined. Increasing *P*_crit_ reduces both the friction force and the COF and increases the normal load threshold below which superlubricity is maintained.

### 4.2 Effect of Particle Radius

One of the key geometrical parameters of the patterned surface is the radius of the particles, i.e., the curvature of the bumps. Thus, it is interesting to explore the effect of the particle radius on the superlubricity of the surface structure. We emphasize that for all simulations, the distance between the tether of the springs of the particles (*p*) is fixed at a constant value of 3 μm (Figure 1). Figure 5a presents the snapshot of the maximum Hertzian contact pressure under a normal load of 2 N with different particle radius. We observe that the macroscale contact radius is only slightly reduced with the increase of particle radius. However, the contact area at each local microscale contact increases, resulting in a reduction of local pressure. Figure 5b illustrates the average maximum contact pressure as a function of normal load with different particle radius. We determine that the surface structure with particles of 5 μm radius experiences lower pressure than that with particles of 3 μm radius. As presented in Figure 5c, we find that the robust transition point occurs at a higher normal load value for a surface with particles of 5 μm radius. The relationship between the transition force (*F*_trans_) and the radius of the microscale particles is presented in Figure 5d. These results highlight the significant influence of microscale bump/particle radius on the superlubricious behavior of the patterned surface. This is consistent with the experimental results,^52^ which found that on laser-patterned surfaces, friction was reduced and superlubric sliding was more robust than on unpatterned surfaces. Furthermore, it suggests the feasibility of engineering a macroscale contact with a specific desired frictional response by tuning the radius of individual microscale bumps/particles.

**Figure 5:**
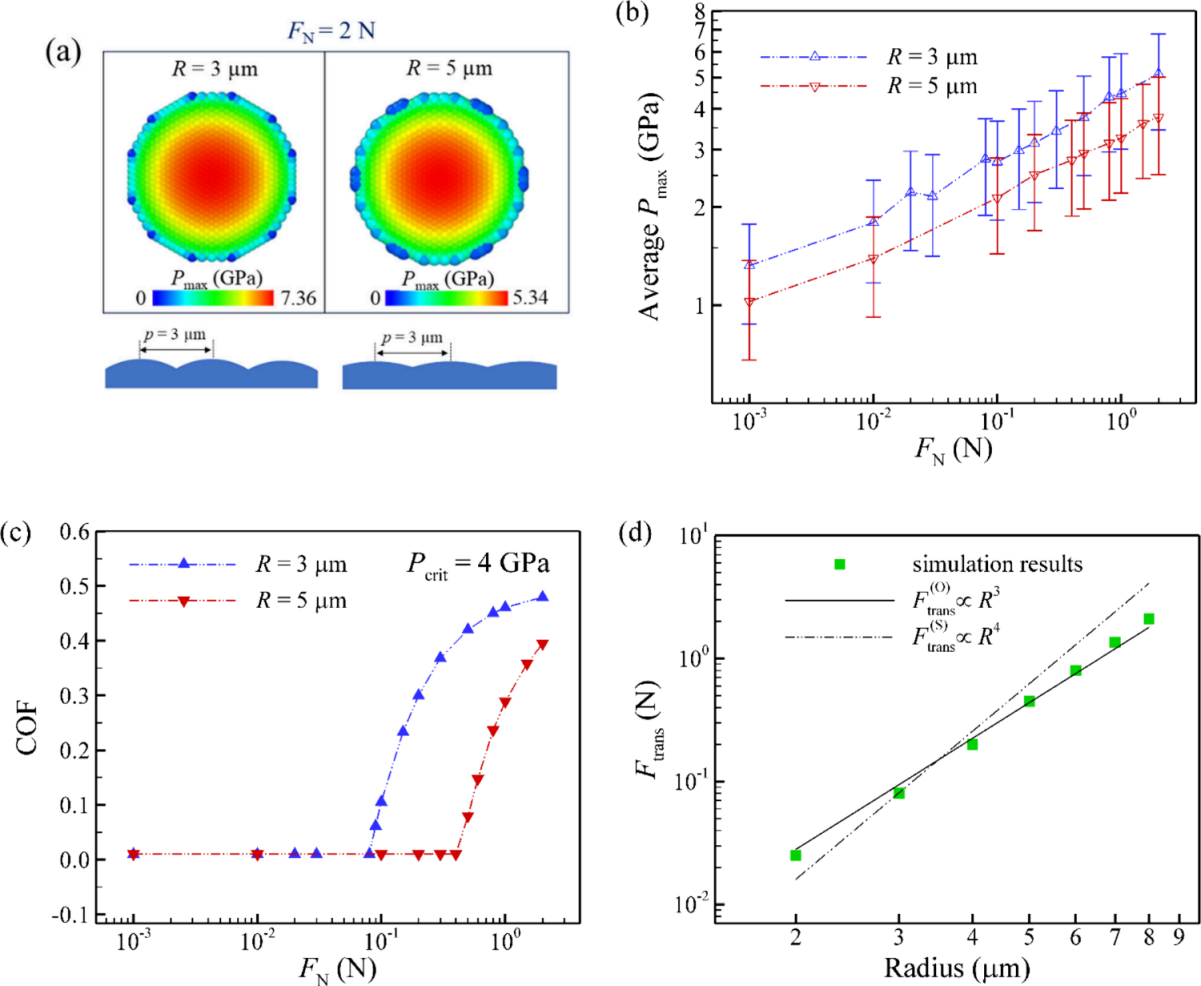
(a) Snapshots of *P*_max_ under a normal load of 2 N with different particle radius. (b) Average *P*_max_ and (c) COF versus normal load with different particle radius. (d) Transition force (*F*_trans_) as a function of particle radius. In this case, *P*_crit_ is set to 4 GPa. An increase in the particle radius reduces the local microscale contact pressure, which improves the robustness of superlubricity.

### 4.3 Comparison between Simulations and Analytical Derivation for Scaling Laws

Turning to this part, we compare the results obtained by simulations to the analytical derivation of the scaling laws. As shown in Figure 3b,c, we observe a good agreement between theoretical predictions (Section 3) and simulation data. Specifically, the dependence of the number of contacts (*N*) and the average *P*_max_ on the normal load lies between the behaviors predicted by the spring mechanism and overlap mechanism. The number of contacts follows a scaling behavior between the power laws with exponents +2/5 and +1/2 (Figure 3b) as described in eqs 18 and 19, while *P*_max_ scales between exponents of +1/6 and +1/5 (Figure 3c) as shown in eqs 14 and 15. Furthermore, the relationship between the transition force and the radius of the microscale particles, shown in Figure 5d, also exhibits intermediate behavior. The scaling of the transition force with particle radius falls between the theoretical predictions for the two mechanisms, corresponding to power-law exponents of +3 and +4 as shown in eqs 16 and 17. These findings suggest that both the spring and the overlap mechanisms are simultaneously active.

### 4.4 Effects of Elastic Parameters

So far, we have shown that in this type of patterned system, the Hertzian contact pressure at a single microscale contact can be a few gigapascals, which can lead to the failure of coating layers. Thus, an important question to answer is how we can reduce the contact pressure to achieve superlubricity. In this section, we explore the significant effects of elasticity of the surface, specifically Young’s modulus and spring stiffness, on the friction response.

First, we present the dependence of the COF on the normal load for structures with three different Young’s moduli in Figure 6a. From this figure, we observe the increase of transition load with lower Young’s modulus of the material. The reason is that when the elastic modulus decreases, the contact area of individual microscale contacts increases, resulting in a lower contact pressure. Furthermore, focusing on the relationship between the average maximum pressure and Young’s modulus, we find that the scaling follows an exponent of +2/3 (Figure 6b), which is consistent with eq 2. The influence of the Young’s modulus on the COF in our system under a normal load of 4 N is shown in Figure 6c. For the high Young’s modulus of 100 GPa, most of the microscale contacts are under high pressure and experience high friction leading to a high macroscopic friction coefficient. If the Young’s modulus of the material is reduced, the fraction of superlubricious microscale contacts increases, resulting in the reduction of the COF. For sufficiently low Young’s modulus, the macroscale contact becomes fully superlubricious. This suggests that a low elastic modulus can help to maintain the macroscale contact under a low friction threshold. Nevertheless, a low elastic constant can also be detrimental to superlubricity. Previous studies have discussed that in an incommensurate system, a stiff solid can prevent elastic instabilities during sliding, preventing breakdown of superlubricity.^37^ In contrast, for soft materials, atoms can rearrange themselves and form commensurate contact, causing high friction.^37,66^ Taking this into account, the patterned surface should be coated with material that has high in-plane elastic constants to preserve the incommensurability of the coatings in the contact. Simultaneously, the bulk material should have a lower elastic modulus to enlarge the contact area at the microscale contact, thereby reducing the local contact pressure.

**Figure 6:**
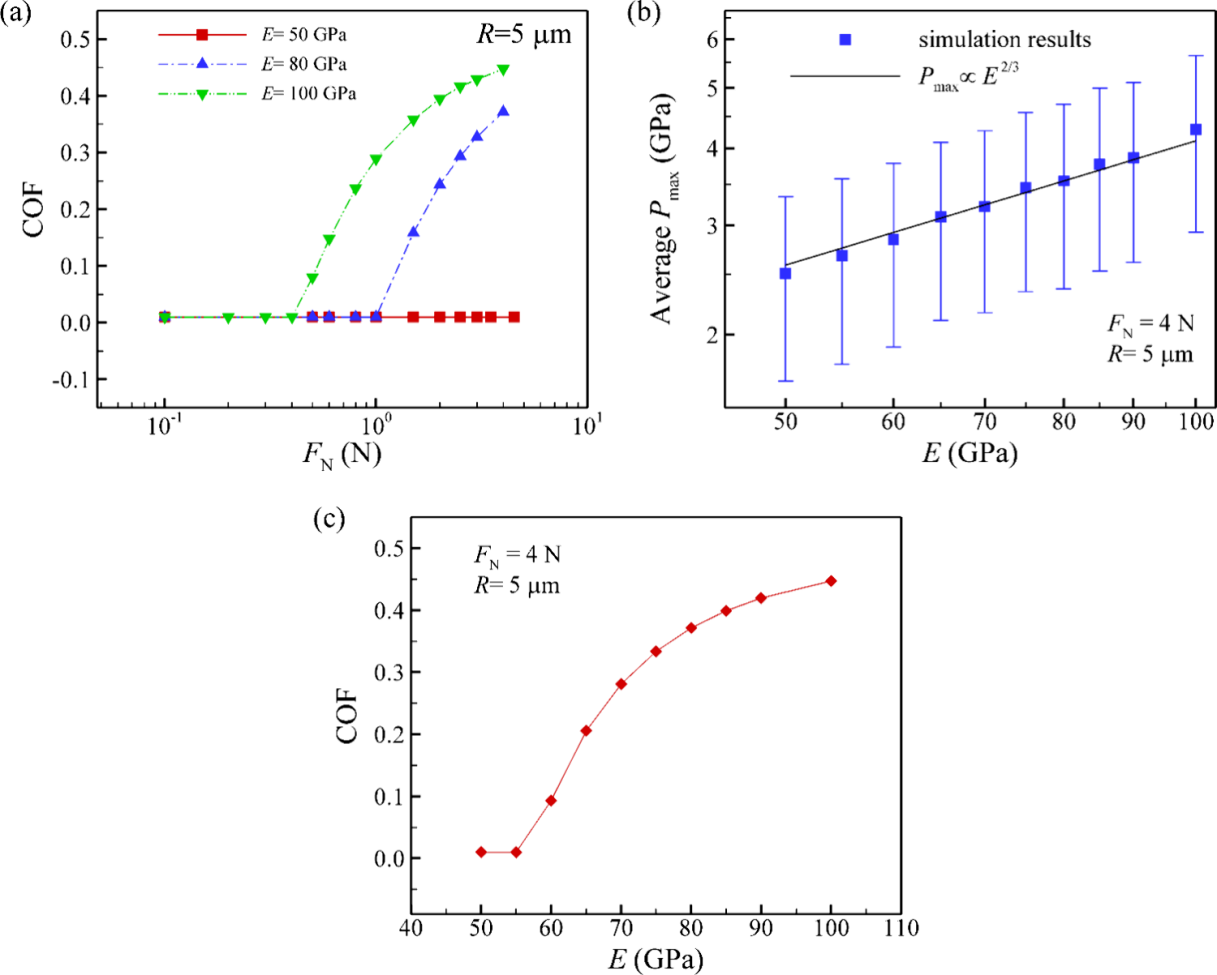
(a) COF and (b) Average *P*_max_ as functions of normal load with different Young’s modulus. (c) COF versus Young’s modulus under normal load of 4 N. A lower elastic modulus can reduce the pressure at microscale contacts and increase the transition load above which superlubricity breaks down.

Next, as described in the simulation method section, each particle is tethered to its initial position by springs, allowing movement in all *x*-, *y*-, and *z*-directions. Since the normal load is applied in the vertical direction, the deformation of the surface is primarily along the *z*-direction. To elucidate the effect of *K*_*z*_ on friction behavior, we performed simulations with a wide range of *K*_*z*_ values from 5 × 10^3^ N/m to 1.1 × 10^5^ N/m. Figure 7a presents the contact pressure at each individual microscale contact under a normal load of 1 N, for varying spring stiffness values. An increase in contact radius and a corresponding reduction in contact pressure are observed with a decreasing spring of *K*_*z*_. As shown in Figure 7b, under the same normal load, in systems with lower spring stiffness, the slider is in contact with a larger number of microscale bumps and particles as opposed to systems with stiffer springs. When the normal load is distributed across more microscale contact, the pressure at individual microscale contact reduces, keeping the macroscale contact in low friction behavior. As presented in Figure 7c, the COF is decreased and the system reaches full superlubricity as the stiffness of the spring is reduced. These results demonstrate that a low elastic constant in the vertical direction is beneficial for reducing friction.

**Figure 7:**
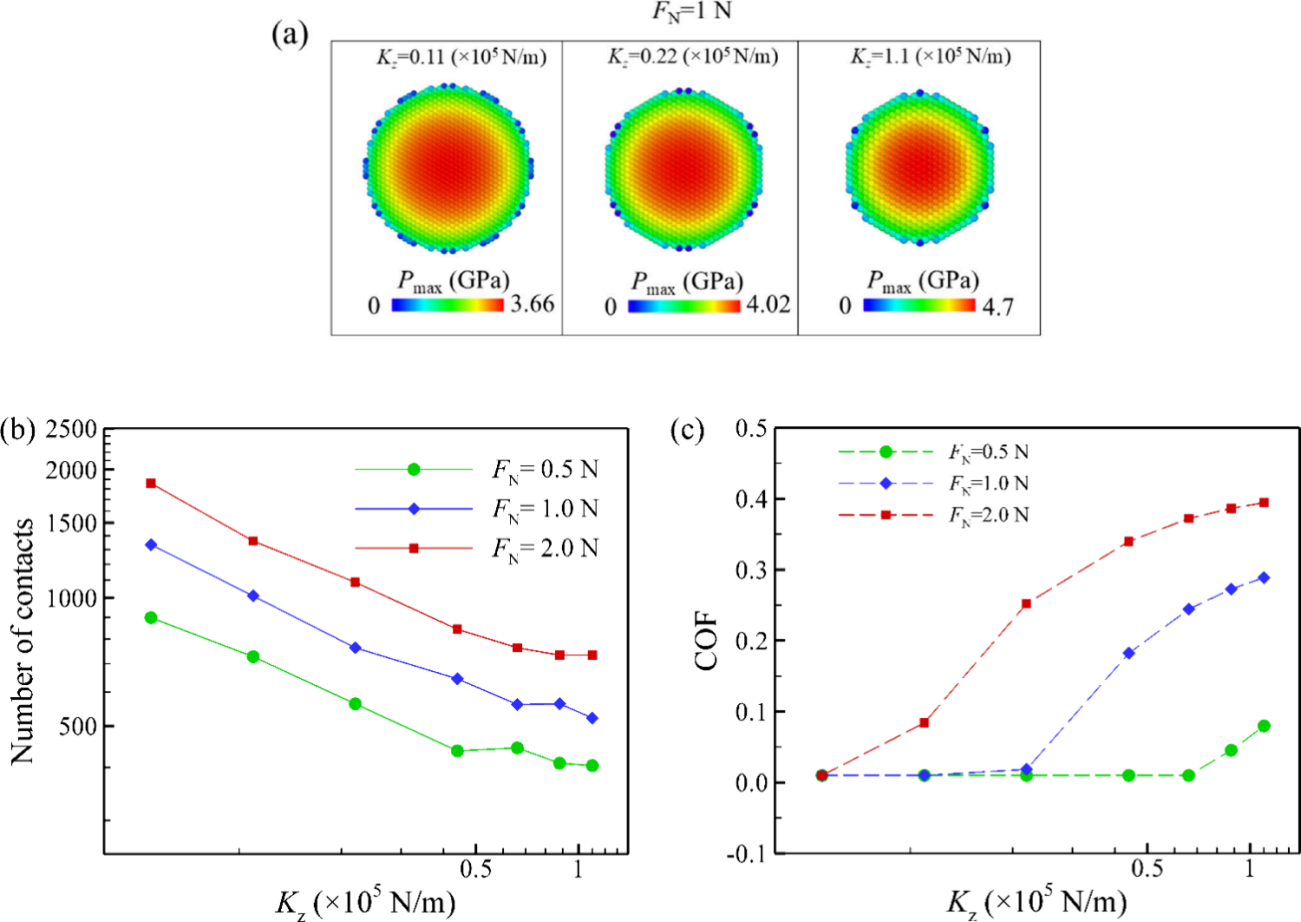
(a) Snapshots of *P*_max_ at each individual microscale contact under a normal load of 1 N with different value of spring stiffness (*K*_*z*_). (b) Number of contacts and (c) COF under 0.5, 1, and 2 N of normal load. A decrease in spring stiffness leads to an increase in the number of contacts and a reduction in the pressure at the microscale contacts.

### 4.5 Effect of Height Variation

No fabrication process is completely flawless, so the microscale bumps on the surface may not all have exactly the same height. We therefore investigate the impact of height variation on the COF. In this analysis, we consider only variations on the micrometer scale, excluding nanoscale asperities on top of the microscale bumps. To examine the effect of height variation, we applied random displacements to all particles in the *z*-direction, with uniform distribution between −Δ*z* and +Δ*z* as illustrated in Figure 8a. Figure 8 shows the relationship between the COF and the normal load for different height variations. To obtain each data point, we performed an average of five different simulations. The height variation leads to fewer contact points (Figure 8b), thereby increasing the local pressure on microcontacts (Figure 8c). Consequently, we observe that the COF is higher for larger variations in height. In fact, we see a significant breakdown in superlubricity already at a relatively small height variation (2% compared with the radius of the particles). Therefore, the height variation should be minimized during the fabrication of the surface.

**Figure 8:**
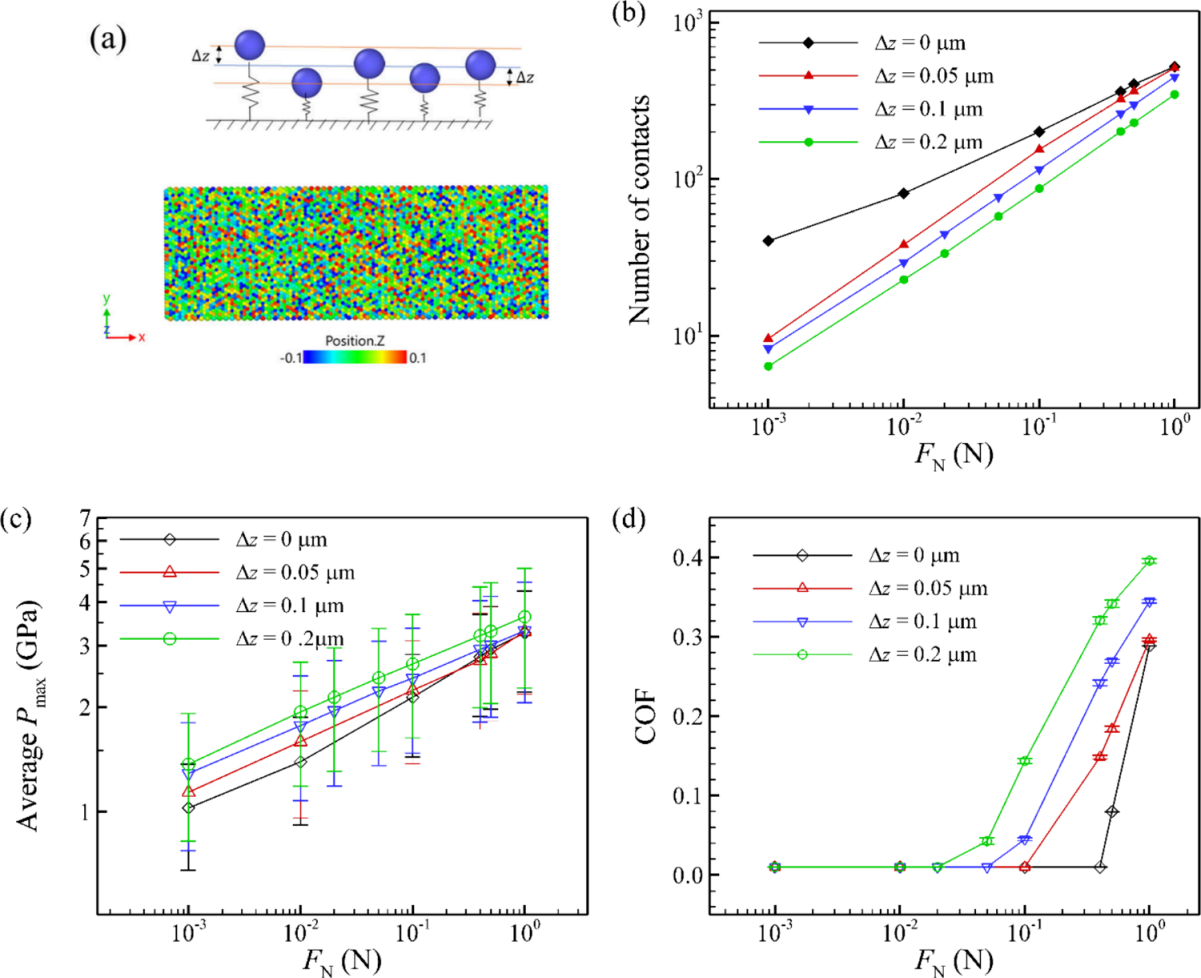
(a) Schematic and snapshot of height variation in which random displacements are applied to all particles in the *z*-direction, with uniform distribution between −Δ*z* and +Δ*z*. (b) Number of contacts, (c) average *P*_max_, and (d) COF as functions of normal load for varying height variations in the surface structure. The radius of the surface particles is 5 μm. Height variations reduce the number of contacts and increase the local contact pressure in the microscopic contacts, which leads to the breakdown of the superlubricity.

## 5 Conclusions

In this work, we investigate the friction behavior of a macroscale patterned surface, which contains multiple microscale contacts, under a macroscopic Hertzian contact. To do that, we perform simulations of granular particles and propose a modified tangential Mindlin contact model to account for the failure of the low-friction coating. The deformation of the patterned surface is analyzed as a combination of overlap and spring compression mechanisms, and friction forces are calculated. We show that the friction, and whether or not the superlubric coating survives, is closely related to the geometry of the interface, similar to what has been observed in experiments on patterned surfaces.^52^ We analytically derive the scaling laws of various quantities, such as the number of microscale contacts, contact pressure, and transition force, with respect to the macroscopic load. Our study highlights the significant dependence of macroscopic friction on several key factors, including the durability of the coating layer, the radius of the microscale bumps, and the elasticity of the patterned surface. We find that the superlubricity breaks down abruptly beyond a particular load. This load is strongly influenced, especially by the critical pressure above which the coating is destroyed. In addition to the critical pressure, the radius of the particles is found to have a significant effect, while other parameters are less impactful, due to having lower scaling exponents. Furthermore, we find that small fabrication defects, such as height variation, can also lead to an early breakdown of the superlubricity. We hope that our results will contribute to the design of novel interfaces and meta-interfaces that can scale up superlubricity for industrial applications, such as mechanical systems, automotive, robotics, and aerospace industry, where superlubricity can be used to reduce friction and enable precise motion between moving parts.

## Supplementary Material



## Notes

Spring stiffness estimation, effect of elastic interaction between particles, verification of modified Mindlin contact model (PDF)
