# Supplementary material for: A Theoretical Study on Friction of Macroscale Patterned Surfaces: Implications for Scaling Up Superlubricity
Source: ACS Appl Mater Interfaces. 2025 Sep 25;17(40):56661–71. doi: 10.1021/acsami.5c16288 (PMC12516681; doi:10.1021/acsami.5c16288)
Supplement: Supplementary file 1 [file am5c16288_si_001.pdf]

Supporting Information

A Theoretical Study on Friction of Macroscale Patterned Surfaces: Implications for Scaling Up Superlubricity

Viet Hung Ho[1], Melisa M. Gianetti[1], Ahmed Uluca[2], Aaron D. Sinnott[2], Bjørn Haugen[1], Graham L. W. Cross[2], and Astrid S. de Wijn[1]

[1] Department of Mechanical and Industrial Engineering, Norwegian University of Science and Technology (NTNU), 7491, Trondheim, Norway.

[2] School of Physics and CRANN, Trinity College Dublin, Dublin 2, Ireland

*Corresponding Author

Email: astrid.dewijn@ntnu.no

Verification of modified Mindlin contact model.

The modified Mindlin contact model is implemented based on the Granular package of LAMMPS (version 23 June 2022). The necessary details required for reproducing the results can be found at the following link: https://github.com/hvhungvn/SSLiP.

To verify the modified Mindlin contact, we performed simulations on sliding between two particles, which have size of 1 μm and 10^5 μm. The friction coefficients used in modified Mindlin contact model are 0.01 and 0.5 for μ_1 and μ_2, respectively. Young's modulus and Poisson's ratio are 100 GPa and 0.3, respectively. For critical pressure (P_{crit}), we examined two different cases of 2 GPa and 4 GPa. Using the Hertzian contact theory, the maximum contact pressure is calculated as shown in equation 2 of the main text

$$P_{\max} = \left(\frac{6 f_N E_{\text{eff}}^2}{\pi^3 R_{\text{eff}}^2} \right)^{1/3}$$

Therefore, the critical force in which COF transits from μ_1 and μ_2 is given as:

$$f_{trans} = \frac{\pi^3 P_{\text{crit}}^3 R_{\text{eff}}^2}{6 E_{\text{eff}}^2} \quad \text{(S1)}$$

For given parameters, theoretical calculations predict the critical force of 3.4 μN and 27.4 μN corresponded to P_{crit} of 2 GPa and 4 GPa, respectively. These calculations are in good agreement with simulation results as shown in Figure S1.

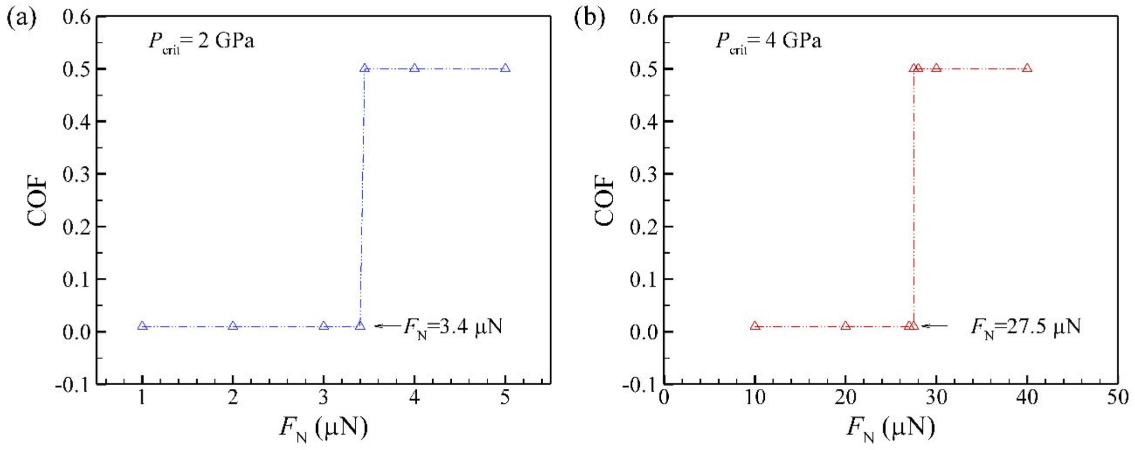

Figure S1. Coefficient of friction (COF) vs normal load with (a) P_{crit}= 2 GPa and (b) P_{crit}= 4 GPa.

Spring stiffness estimation.

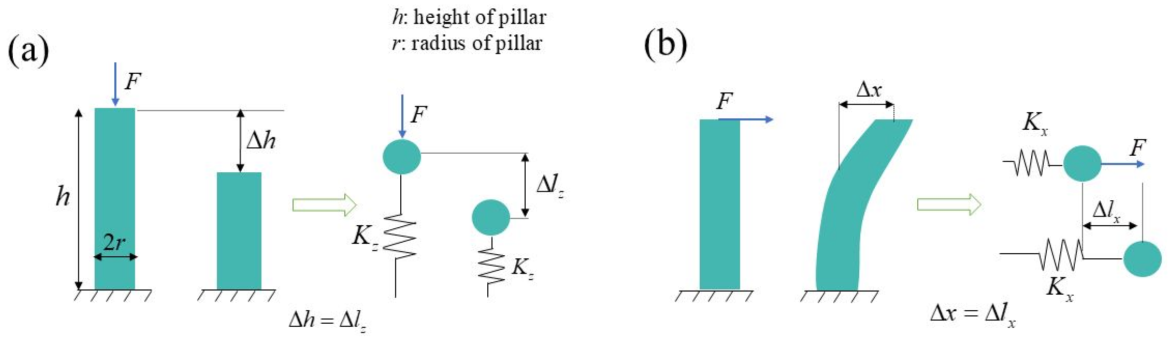

Figure S2. Model of a cylindrical pillar under loading. (a) Normal and (b) lateral loading. The deformation of the pillar is represented by granular particles and springs, as described in Section 2 of the main text.

As mentioned in the main text, each particle on the surface is tethered to its initial position by springs in the *x*-, *y*-, and *z*- directions. To estimate the spring stiffness values, we consider a simplified model in which each particle is treated as a cylindrical pillar with a radius r and height h. The deformation of the pillar under normal and lateral loading is then modeled using spherical particles and springs K_x, K_y and K_z in numerical simulation. The spring stiffnesses values are subsequently estimated as follows:

$$K_x = K_y = \frac{3\pi E r^4}{4h^3} \tag{S2}$$

$$K_z = \frac{\pi E r^2}{h} \tag{S3}$$

with E is the Young's modulus of material. Given a radius (r) of 0.9 µm, height (h) of 2.3 µm, and Young's modulus of 100 GPa, the spring stiffness values are estimated as 1.27×10^4 N/m for the in-plane K_x and K_y, and 1.1×10^5 N/m for K_z. These stiffness values are affected by geometry of the pillar and may vary with changes in its dimensions.

Effect of elastic interaction between particles.

In this section we examined the effect of elastic interaction between particles on friction behaviour. To address it, we have performed additional simulations in which neighboring particles were connected by springs (K_p) with varying stiffness, as illustrated in Figure S3. The results show that the coefficient of friction remains unchanged across a wide range of spring stiffness, indicating that the effect of elastic interactions between particles is negligible.

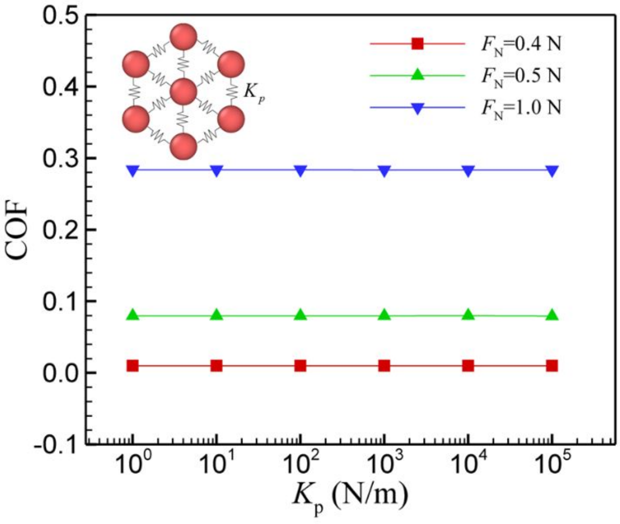

Figure S3. The coefficients of friction as function of spring stiffness K_p at different applied normal load.
